# Wearable and Portable Devices for Acquisition of Cardiac Signals while Practicing Sport: A Scoping Review

**DOI:** 10.3390/s23063350

**Published:** 2023-03-22

**Authors:** Sofia Romagnoli, Francesca Ripanti, Micaela Morettini, Laura Burattini, Agnese Sbrollini

**Affiliations:** Department of Information Engineering, Università Politecnica delle Marche, 60131 Ancona, Italy; s.romagnoli@pm.univpm.it (S.R.); s1101456@studenti.univpm.it (F.R.); m.morettini@univpm.it (M.M.); a.sbrollini@staff.univpm.it (A.S.)

**Keywords:** wearable, portable, sensor, device, heart rate, electrocardiography, monitoring, sport

## Abstract

Wearable and portable devices capable of acquiring cardiac signals are at the frontier of the sport industry. They are becoming increasingly popular for monitoring physiological parameters while practicing sport, given the advances in miniaturized technologies, powerful data, and signal processing applications. Data and signals acquired by these devices are increasingly used to monitor athletes’ performances and thus to define risk indices for sport-related cardiac diseases, such as sudden cardiac death. This scoping review investigated commercial wearable and portable devices employed for cardiac signal monitoring during sport activity. A systematic search of the literature was conducted on PubMed, Scopus, and Web of Science. After study selection, a total of 35 studies were included in the review. The studies were categorized based on the application of wearable or portable devices in (1) validation studies, (2) clinical studies, and (3) development studies. The analysis revealed that standardized protocols for validating these technologies are necessary. Indeed, results obtained from the validation studies turned out to be heterogeneous and scarcely comparable, since the metrological characteristics reported were different. Moreover, the validation of several devices was carried out during different sport activities. Finally, results from clinical studies highlighted that wearable devices are crucial to improve athletes’ performance and to prevent adverse cardiovascular events.

## 1. Introduction

Over the last decade, wearable and portable devices for cardiac monitoring have become increasingly popular, as they are relatively inexpensive and user-friendly. Miniaturized technologies and powerful signal processing applications make them a noninvasive, cheap, and time-efficient tool for cardiac monitoring while playing sport outside a clinically controlled environment [1,2,3,4].

Wearable devices are designed to be worn on different body locations for noninvasive sensing of an individual’s parameters without interrupting or restricting the user’s movements. Portable devices are designed to monitor cardiac conditions more easily than traditional monitors, being small and lightweight. On a sport field, portable devices may be useful in documenting and contributing to diagnosis of exercise-induced arrhythmias [5,6].

Electrocardiography (ECG) and heart rate (HR) are the main signals used to evaluate cardiac status during sport [7]. The ECG represents cardiac electrical activity and HR is the number of times the heart beats within a one-minute period [8]. Usually, HR is derived from the time intervals among consecutive heart beats detectable from the ECG or the photoplethysmogram (PPG), which represent the peripheral effect of the heart pulse [9]. Thus, the sensing modality mainly used for cardiac signal acquisition are electrodes (wet, dry, and capacitive), able to acquire the ECG, or optical sensors, able to acquire the PPG [9,10]. A recent development is based on sensing the mechanical activity of the heart [9]. Mechanocardiography consists in detecting organ motion caused by the heart beat by measuring displacements and vibrations of the body surface caused by the pulse wave traveling through the body [9].

Some sensors have already been integrated into standard clinical practice, whereas some others exist for use in consumer health and medical research [2,4,10]. During sport activity, wearable and portable devices are commonly used to reliably acquire cardiac functionality and to provide useful clinical information on an athlete’s health status [4,11,12]. Information gathered from these devices may be used by coaches to optimize athlete training and performance and by clinicians to monitor athlete health and evaluate the cardiovascular risk under physical/psychological stress [3,4,13].

These technologies cover a big area of the consumer wearable market and lead development trends in sport industry [10,14,15]. Wearable devices were the top trend in an electronic survey of health and fitness trends by ACSM’s *Health & Fitness Journal* for 2022, and they have been estimated to be a $100 billion industry in the US [14]. Market research forecasts a grow in the sport and fitness industry with heavy future investment in terms of industrial research, with the aim to improve the sensors in terms of flexibility, motion, and smart textiles [10,14,16]. New innovations further include the reliable estimation of blood pressure, oxygen saturation, body temperature, and respiratory rate [14,17,18,19]. Of note, optical sensory components will lead revenue for wearable devices [10].

The present scoping review investigated the commercial wearable and portable devices acquiring cardiac signals that were/are used in the sport research field. The aim was to define the trends in wearable and portable devices usage and to identify research gaps in their application to the sport field.

## 2. Materials and Methods

The literature search and method reporting performed here followed the PRISMA extension for scoping reviews (PRISMA-ScR) [20].

### 2.1. Literature Search Strategy

A systematic literature search was conducted on three electronic bibliographic databases: PubMed, Scopus, and Web of Science. The roots “athlet” and “sport” were used to search for studies in the sport field. The roots “sensor”, “electronic” and “device” accompanied by the adjectives “wearable” and “portable” were used to search for studies on wearable and portable monitoring systems. The keyword “heart rate” and the root “electrocardio” were used to search for studies on cardiac signals. The search terms were organized into three concepts:

1. athlet*, sport*;
2. wearable, portable, sensor*, electronic*, device*;
3. heart rate, electrocardio*.

Terms within the first and third concepts were combined with the Boolean operator “OR”, within the second concept the terms “wearable” and “portable” were combined with the Boolean operator “OR”, and the terms “sensor*,” “electronic*,” and “device” were combined with the Boolean operator “OR” and between them were combined with the Boolean operator “AND”. Then, concepts were combined with the Boolean operator “AND”.

“Title” and “Abstract” were used as limits for the search field, English and Spanish as limits to filter language, “2022” as maximum limit to filter publication years, and “Review” as exclusion criterion for type of document (thus, all other document types were included). The search query is reported in Supplementary Materials, file name “Search_Query.pdf”.

### 2.2. Selection of Studies

Obtained documents were imported into the Mendeley reference management system for duplicate removal. Eligibility criteria for title, abstract, and full-text screening and selection were:

1. studies focusing on commercially available wearable or portable devices able to acquire cardiac signals, namely, ECG and HR;
2. studies proposing wearable and portable devices used during sport practice;
3. studies considering populations of athletes, recruited without limits on sport level, from recreational to elite athletes.

Documents for which the full text was not available were excluded.

### 2.3. Data Charting and Synthesis

A data-charting form was jointly developed by two reviewers to determine which variables to extract. The two reviewers independently charted the data, and discrepancies were resolved after joint discussion.

Studies were classified as validation studies if aiming to validate a device, clinical studies if aiming to evaluate the pathophysiological states and/or performances of athletes, and development studies if aiming to design and validate algorithms and/or to create databases. Validation studies were described in terms of validated device, reference devices, acquired signal, sport activity, population, and validation results. Clinical studies were described in terms of device, acquired signals, sport activity, population and aim of device application. Development studies were described in terms of device, acquired signal, sport activity, population and aim of device application. Data were synthesized in tables.

Each device was described in terms of acquired signal (ECG and/or HR), sensor tech (wet electrode, dry electrode, capacitive electrode, optical), wear location, target user (athlete, coach, clinician), real-time output, other integrated sensors, feedback, associated app, and clinical approval (such as FDA approval). Specification of wearable and portable devices were retrieved from technical and user manuals or in the manufacturer website. The sources are reported in Supplementary Materials, file name “Specification_Device_Sources.pdf”. Clinical approval was checked on the FDA website https://www.accessdata.fda.gov/scripts/cdrh/cfdocs/cfpmn/pmn.cfm (last access on 15 February 2023).

## 3. Results

**Figure 1:**
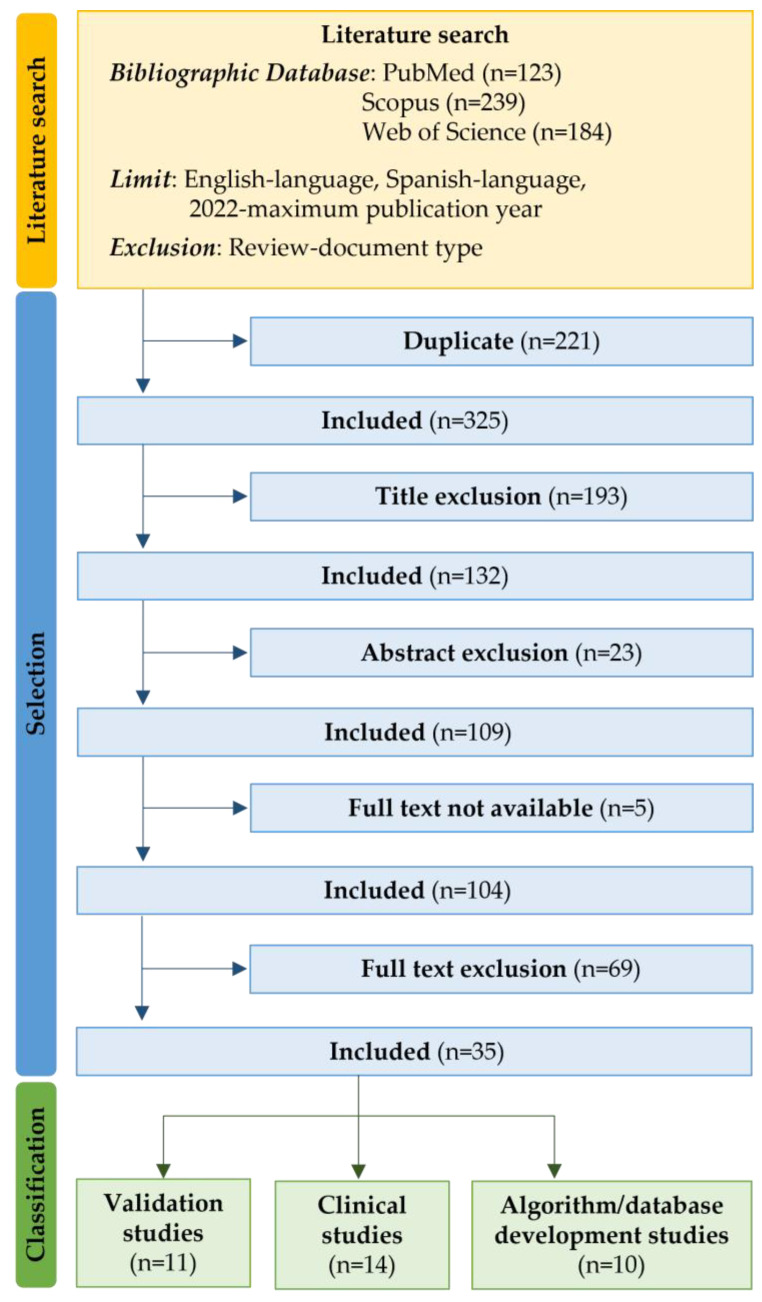
Flowchart of systematic literature search study selection and classification.

Overall, 546 studies were identified in the bibliographic databases. Of these, 221 were duplicates, so 325 were left for further analysis. After title, abstract and full-text screening based on eligibility criteria, 35 studies were selected. Figure 1 depicts the entire process of the systematic literature search study selection and classification. The selected studies consisted of 26 journal papers, 8 conference proceedings, and 1 book chapter. Their classification provided 11 validation studies (Table 1), 14 clinical studies (Table 2), and 10 development studies (Table 3). Despite both English and Spanish languages being considered, all papers were written in English.

From 2011 to 2022, 38 different commercial wearable and portable devices were employed for research purposes: 23 wrist-worn, 5 chest straps, 2 forearm bands, 2 mobile ECG recorders, 1 biometric shirt, 3 bra, 1 earbud and 1 ring. Table 4 reports each device along with its characteristics: acquired signal (ECG and/or HR), sensor tech, wear location, target user, real-time output, other integrated sensor, feedback, associated app, and clinical approval (FDA). The most studied brand was Polar and the most studied sport running.

## 4. Discussion

In the last few decades, the use of wearable and portable devices that allow real-time acquisition of vital parameters has increased significantly. The purpose of this study was to investigate the commercial wearable and portable devices acquiring cardiac signals, ECG and HR used in sport.

After the literature search and review, 35 studies were included. Review-type documents were excluded because they are secondary studies. Moreover, quality and scope vary widely and thus can influence the conclusions drawn. A systematic literature search was conducted based on the generic terms in the search string without a specific name of device or sport, leading to the exclusion of some articles from the search because their title or abstract stated the specific name of the device and sport.

**Table 1:** Validation studies characterized by the validated device, the reference device, the acquired signal (ECG and/or HR), the practiced sport activity, the population characteristics and the validation results. Devices are reported with their commercial name and the population characterized in terms of sex (male/female), age, ethnicity and BMI. If not, present height and weight are reported. Information not available is reported as “-”.

Ref.	Validated Device	Reference Device	Acquired Signal	Sport Activity	Population	Validation Results
[21]	Polar Vantage V2	Polar H10 chest strap	HR	Swimming	10 healthy athletic subjects SEX: - AGE: 17.0 ± 3.0 years BMI: 19.80 ± 1.21 kg/m^2^	*Rest dry condition:* μ = −5 bpm; 2σ = ±19 bpm; r = 0.32; CI95% = [–24, 13] bpm; MAPE = 7.32% *Active dry condition:* μ = −4 bpm; 2σ = ±24 bpm; r = 0.83; CI95% = [−28, 19] bpm; MAPE = 8.29% *Rest in water:* μ = −4 bpm; 2σ = ±28 bpm; r = 0.62; CI95% = [−32, 24] bpm; MAPE = 10.37% *Swim in water:* μ = −18 bpm; 2σ = ±68 bpm; r = 0.2; CI95% = [−84, 49] bpm; MAPE = 29.78%
	Garmin Venu Sq					*Rest dry condition:* μ = −1 bpm, 2σ = ±16 bpm; r = 0.65; CI95% = [−17, 15] bpm; MAPE = 4.83% *Activity dry condition:* μ = −1 bpm; 2σ = ±12 bpm; r = 0.32; CI95% = [−52, 28] bpm; MAPE = 17.32% *Rest in water:* μ = −12 bpm; 2σ = ±41 bpm; r = 0.32; CI95% = [−52, 28] bpm; MAPE = 17.32% *Swim in water:* μ = −57 bpm; 2σ = ±68 bpm; r = 0.13 CI95% = [−124, 10] bpm; MAPE = 58.94%
[22]	Kardia 6L AliveCor	12-lead ECG	ECG	Cricket	30 healthy athletes SEX: 17/13 AGE: mean 18.9 years WEIGHT: - HEIGHT: - BMI: -	Mean difference _HR_ = 3 ± 9 bpm Mean difference _QT_ = −18 ± 14 ms Mean difference _QTc_ = −10 ± 18 ms Mean difference _QRS_ = −3 ± 7 ms Mean difference _PR_ = −6 ± 8 ms
[23]	Polar Ignite sport watch	Polar H10 chest strap	HR	Specific training program	11 recreational athletes SEX: 6/5 AGE: 21.73 ± 1.49 years BMI: 23.41 ± 2.99 kg/m^2^	r = 0.714 ICC = 0.817
[24]	Polar H7 chest-strap	3-lead ECG	HR	Running	50 healthy athletic subjects SEX: 34/16 AGE: 29.5 ± 9.3 years BMI: 22.8 ± 2.4 kg/m^2^	r_c_ = 98
	Apple Watch III					r_c_ = 98
	Fitbit Ionic					r_c_ = 89
	Garmin Vivosmart HR					r_c_ = 89
	TomTom Spark 3					r_c_ = 89
[25]	Garmin Fenix 5	Polar H7 chest strap	HR	Trail running	21 healthy subjects SEX: 11/10 AGE: 31.0 ± 11.0 years WEIGHT: 75.6 ± 12.9 kg HEIGHT: 173.0 ± 6.9 cm	MAPE = 13%; LOA = [−32, 162]; r_c_ = 0.32
	Jabra Elite Sport Earbuds					MAPE = 23%; LOA = [−464, 503]; r_c_ = 0.38
	Motiv Ring					MAPE = 16%; LOA = [−52, 96]; r_c_ = 0.29
	Scosche Rhythm+					MAPE = 6%; LOA = [−114, 120]; r_c_ = 0.79
	Suunto Spartan Sport watch					MAPE = 2%; LOA = [−62, 61]; r_c_ = 0.96
[26]	Polar H10 chest strap	12-lead ECG	HR	Cycling incremental exercise	25 recreational athletes SEX: 14/11 AGE: *male* 40.0 ± 14.0 years *female* 34.0 ± 10.0 years WEIGHT: *male* 82.2 ± 4.8 kg *female* 67.8 ± 9.5 kg HEIGHT: *male* 178.1 ± 9.0 cm *female* 169.1 ± 4.3 cm	*Rest pre-exercise:* r = 0.95; r_c_ = 0.95; ICC_3,1_ = 0.95; *Rest post-exercise:* r = 0.86; r_c_ = 0.84; ICC_3,1_ = 0.85 *Incremental exercise:* r > 0.93; r_c_ > 0.93; ICC_3,1_ > 0.93
[27]	Polar H7 chest strap	12-lead ECG	HR	Aerobic exercise	50 healthy subjects SEX: 23/27 AGE: 38.0 ± 12.0 years BMI: 25.0 ± 3.5 kg/m^2^	r_c_ = 0.996
	Scosche Rhythm+					r_c_ = 0.75
	Apple Watch I					r_c_ = 0.92
	Fitbit Blaze					r_c_ = 0.67
	Garmin Forerunner 235					r_c_ = 0.81
	TomTom Spark Cardio					r_c_ = 0.83
[28]	Polar OH1	Polar H10 chest strap	HR	Light, moderate, vigorous, and sprint-based exercise	20 healthy subjects SEX: 11/9 AGE: 40.0 ± 10.0 years WEIGHT: 71.6 ± 11.0 kg HEIGHT: 173.0 ± 10.0 cm	Mean bias = −1 bpm; LOA = [−20, 19] bpm; MAPE = 0.4%; r = 0.957; CI95% = [0.956, 0.958] bpm
	Fitbit Charge 3					Mean bias = −7 bpm; LOA = [−46, 33] bpm; MAPE = −4%; r = 0.807; CI95% = [0.804, 0.811] bpm
[29]	Polar Vantage M	3 leads plus V5 ECG	HR	Treadmill exercises (Bruce protocol)	29 healthy subjects SEX: 16/13 AGE: *male* 26.25 ± 3.17 years *female* 26.00 ± 3.85 years BMI: *male* 25.54 ± 2.54 kg/m^2^ *female* 22.50 ± 2.07 kg/m^2^	*Stage 0:* Test–retest reliability = 0.42; CI95% = [−0.27, 0.73] bpm *Stage 1:* Test–retest reliability = 0.78; CI95% = [0.54, 0.90] bpm *Stage 2:* Test–retest reliability = 0.78; CI95% = [0.54, 0.90] bpm *Stage 3:* Test–retest reliability = 0.68; CI95% = [0.32, 0.85] bpm *Stage 4:* Test–retest reliability = 0.58; CI95% = [0.14, 0.80] bpm *Stage 5:* Test–retest reliability = 0.92; % = [0.79, 0.97] bpm
[30]	PulseOn	Polar V800 HR monitor	HR	Running	24 healthy subjects SEX: 13/11 AGE: 36.2 ± 8.2 years BMI: 22.7 ± 1.9 kg/m^2^	MAPE = 1.9%
[31]	Adidas Smart sports bra	Polar H7 chest strap	HR	Walking Running	24 healthy subjects SEX: 0/24 AGE: 22.2 ± 5.8 years WEIGHT: 71.2 ± 14.4 kg HEIGHT: 174.6 ± 9.9 cm	*Valid at rest* ICC = 0.79; MAPE = 4.5%; LoA = [−8, 8]
	Sensoria fitness sports bra + HRM					*Valid at rest and walking* ICC = 0.96; MAPE = 1.9%; LoA = [−19, 19]
	Berlei sports bra					*Valid at rest, walking and running* ICC = 0.99; MAPE = 0.66%; LoA = [−15, 12]

ICC = interclass correlation coefficient; μ = accuracy; 2σ = precision; CI95% = 95% confidence interval; MAPE = mean average percentage error; r = Pearson correlation coefficient, LOA = limit of agreement; r_c_ = Lin’s concordance coefficient.

**Table 2:** Clinical studies characterized by the used wearable or portable device, the acquired signal (ECG and/or HR), the practiced sport activity, the population characteristics and the aim of device application. Devices are reported with their commercial name and the population characterized in terms of sex (male/female), age, and BMI. If not, present height and weight are reported. Information not available is reported as “-”.

Ref.	Device	Acquired Signal	Sport Activity	Population	Aim of Device Application
[32]	Polar S810i	HR	Speed skating marathon	1 highly trained athlete SEX: 1/0 AGE: 20.0 years WEIGHT: 73.4 kg HEIGHT: 178.0 cm	Monitoring HR (along with oxygen uptake and speed) to quantify and describe the exercise intensity
[33]	Polar S810	HR	Badminton	7 professional players SEX: 3/4 AGE: 16.9 ± 2.1 years WEIGHT: 62.8 ± 9.2 kg HEIGHT: 171.0 ± 9.0 cm	To compare cardiorespiratory and metabolic responses during on-court and simulated badminton rally at different intensities
[34]	Polar Team Pro sensor	HR	Basketball	10 athletes SEX: 0/10 AGE: 19.8 ± 1.3 years WEIGHT: 78. 1 ± 5.8 kg HEIGHT: 179.1 ± 6.0 cm	Assess HR responses and time spent in 5 different HR zones to monitor NCAA division I women’s basketball athletes throughout each 4-quarter game
[35]	Polar Team Pro sensor	HR	Basketball	13 athletes SEX: 0/13 AGE: 19.6 ± 1.3 years WEIGHT: 77.7 ± 5.6 kg HEIGHT: 179.4 ± 5.6 cm	Monitoring HR and HR zones (along with VO_2max_, body weight training load) to assess factors that contribute to countermovement jump performance
[36]	Polar Team Pro sensor	HR	Football	20 players SEX: - AGE: <19 years WEIGHT: - HEIGHT: - BMI: -	To provide an understanding of how Polar Team Pro is being implemented in competitive football training process, in terms of evaluation and monitoring the official games’ parameters
[37]	Polar Team Pro sensor	HR	Soccer, Basketball, Volleyball	64 collegiate athletes SEX: 64/0 AGE: 20.7 ± 1.9 years WEIGHT: 62.6 ± 6.1 kg HEIGHT: 171.3 ± 6.2 cm	To quantify the physical and physiological response during three widely practiced leisure-time sports using the GPS and HR monitors
[38]	Polar Team Pro sensor	HR	Basketball	11 athletes SEX: 0/11 AGE: 19.6 ± 1.4 years WEIGHT: 78.5 ± 5.7 kg HEIGHT: 179.7 ± 6.0 cm	Measure HR and its peaks to assess caloric expenditure throughout 31 games
[39]	Polar H10 chest strap	HR	Running, Basketball, Badminton	14 recreational athletes SEX: 14/0 AGE: 24.9 ± 2.4 years WEIGHT: 74.6 ± 6.9 kg HEIGHT: 177.0 ± 4.0 cm	To quantify the strength of the relationship between the percentage of HR reserve and two acceleration-based intensity metrics under three intensity conditions
[40]	Polar chest belt *	HR	Running in hilly terrain	17 elite athletes SEX: 13/4 AGE: *male* 29.0 ± 4.0 years *female* 30.0 ± 8.0 years BMI: *male* 71.9 ± 5.6 kg/m^2^ *female 59*.9 ± 4.8 kg/m^2^	To investigate cardiorespiratory and metabolic response. To compare whether HR adequately reflects the exercise intensity or whether the tissue saturation index could provide a more accurate measure
[41]	Polar H10 chest strap	HR	Walking, Running	120 healthy subjects *30 sedentary subjects* SEX: 12/18 AGE: 21.9 ± 1.9 years BMI: 23.7 ± 3.5 kg/m^2^ *30 Exercise habit group* SEX: 14/16 AGE: 21.7 ± 1.6 years BMI: 23.1 ± 3.3 kg/m^2^ *30 Non-endurance group* SEX: 17/13 AGE: 21.1 ± 1.7 years BMI: 23.3 ± 4.7 kg/m^2^ *30 Endurance group* SEX: 19/11 AGE: 20.9 ± 1.7 years BMI: 20.8 ± 2.1 kg/m^2^	To include the HR reserve as a compensatory parameter for physical intensity
[42]	BioHarness 3.0 Zephyr	ECG HR	Running, Soccer, Cycling	20 healthy subjects 10 sedentary subjects SEX: - AGE: 26 [25–31] years WEIGHT: 73 [70–78] kg HEIGHT: 179 [167–183] cm 10 amateur athletes SEX: - AGE: 28 [24–36] years WEIGHT: 69 [57–75] kg HEIGHT: 173 [165–185] cm	To develop and test a low-cost, large-scale procedure for HR and HRV monitoring from signals obtained using comfortable wearable sensors, finalized to evaluate the health status of an athlete besides his/her performance level
[43]	BioHarness 3.0 Zephyr	ECG	Basket, Cycling, Fitness, Jogging, Middle-distance running, Tennis, CrossFit	51 athletes SEX: 38/13 AGE: 29.0 ± 11.0 years WEIGHT: 68.0 ± 10.0 kg HEIGHT: 175.0 ± 6.0 cm	To provide normal reference values of HR and electrocardiographic features for the pre-exercise phase to support large-scale prevention programs fighting sport-related sudden cardiac death
[44]	Hexoskin shirt	HR	Badminton	1 elite badminton player SEX: - AGE: - WEIGHT: - HEIGHT: - BMI: -	To investigate of the relationship between movement accuracy and HR
[45]	Kardia 6L AliveCor	ECG	Cricket, Running	6 amateur and elite athletes SEX: 6/0 AGE: 28 [28–38] years WEIGHT: - HEIGHT: - BMI: -	To highlights the use of the device in aiding the diagnosis of arrhythmias in the setting of exercise-related symptoms in athletes through smartphone ECG

GPS = Global Positioning System; NCAA = National Collegiate Athletic Association. * Chest belt version is not specified.

**Table 3:** Development studies characterized by the used wearable or portable device, the acquired signal (ECG and/or HR), the practiced sport activity, the population characteristics and the aim of device application. Devices are reported with their commercial name and the population characterized in terms of sex (male/female), age, and BMI. If not, present height and weight are reported. Information not available is reported as “-”.

Ref.	Device	Acquired Signal	Sport Activity	Population	Aim of Device Application
[46]	Polar H10 chest strap	ECG, HR	Running	31 athletes SEX: 22/9 AGE: 34.0 ± 10.0 years WEIGHT: 70.0 ± 12.0 kg HEIGHT: 170.0 ± 9.0 cm	To assess the performance of breathing rate estimation algorithm using HR acquired with a chest belt during physical activities
[47]	Polar T31^TM^ Coded band	HR	Swimming	10 federated athletes SEX: - AGE: [15,16,17] years WEIGHT: - HEIGHT: - BMI: -	To propose a data analytics system (including pre-processing of raw signals, feature representation, online recognition of the swimming style and turns, and post-analysis of the performance for coaching decision support) for swimmer performance
[48]	Polar T31^TM^ Coded band	HR	Swimming	10 federated athletes SEX: - AGE: [15,16,17] years WEIGHT: - HEIGHT: - BMI: -	To propose a system that allows the technical staff to monitor and analyze the swimmer by integrating inertial data and bio-signal in real time
[49]	BioHarness 3.0 Zephyr	ECG, HR	Aerial silks, Running, Tennis	10 athletes SEX: 3/7 AGE: 27.0 ± 11.0 years WEIGHT: - HEIGHT: -	To propose an application, CaRiSMA 1.0, analyzing the ECG and HR acquired during a training session and provides intuitive graphical outputs on resting QTc and on exercise HR
[50]	BioHarness 3.0 Zephyr	ECG and automatically computes HR series	Aerial silks, Basketball, CrossFit, Fitness, Jogging, Middle-distance running, Running, Soccer, Tennis, Zumba	81 athletes SEX: 53/28 AGE: 30.0 ± 13.0 years WEIGHT: 71.0 ± 21.0 kg HEIGHT: 170.0 ± 30.0 cm	To provide a database of 126 cardiorespiratory data (demographic info—cardiorespiratory signals and training notes) acquired from 81 subjects while practicing 10 different sports
[51]	BioHarness 3.0 Zephyr	HR	Soccer	21 players SEX: 0/21 AGE: - WEIGHT: - HEIGHT: - BMI: -	To present a predictive analytics framework for analyzing and predicting soccer players’ performance data (HR and speed parameters)
[52]	BioHarness 3.0 Zephyr	HR	Middle-distance running, Jogging	17 athletes SEX: 15/2 AGE: 35.0 ± 14.0 years WEIGHT: - HEIGHT: - BMI: -	To develop an algorithm for automatic detection of training phases in HR series to boost signal processing for athletic cardiovascular monitoring with wearable devices
[53]	Samsung Galaxy Watch 3	HR	High intensity workout	98 athletes SEX: 47/51 AGE: 33.00 ± 8.46 years BMI: 22.78 ± 2.92 kg/m^2^	To develop an ultra-lightweight framework for a precise real-time HR monitoring during the high intensity physical exercises
[54]	Garmin Forerunner 305	HR	Aerobic activity	8 athletes SEX: 7/1 AGE: 27.88 ± 2.17 years BMI: 23.68 ± 4.13 kg/m^2^	To present a system able to estimate the intensity of activities and to identify physical activity and posture
[55]	Hexoskin shirt	HR	Climbing	1 athlete SEX: - AGE: - WEIGHT: - HEIGHT: - BMI: -	To examine time-resolved sensor-based measurements of multiple biometrics at different micro locations along a climbing route

**Table 4:** Commercial wearable and portable devices characterized by acquired signal (ECG and/or HR), sensor tech, wear location, target user, real-time output, other integrated sensor, feedback, associated app, clinical approval. Information not available is reported as “-”.

Device	Acquired Signal	Sensor Tech	Wear Location	Target User	Real time Output	Other integrated Sensor	Feedback	Associated App	Clinical Approval
Apple Watch I	HR	Optical	wrist	athlete	HR on watch	Accelerometer Gyroscope	Irregular cardiac rhythm notification	Apple watch app Health app	NO
Apple Watch III	HR	Optical	wrist	athlete	HR on watch	GPS/GLONASS/Galileo Accelerometer Gyroscope Barometric altimeter	Irregular cardiac rhythm notification	Apple watch app Health app	NO
BioHarness 3.0 Zephyr	ECG HR	Capacitive electrode	chest	athlete	-	3-axis accelerometer Breathing sensor Thermistor	Subject status indication	Bluetooth BioHerness test app	NO
Fitbit Blaze	HR	Optical	wrist	athlete	HR on watch	MEMS 3-axis accelerometer Barometric altimeter	HR zones	Fitbit app	NO
Fitbit Charge 3	HR	Optical	wrist	athlete	HR on watch	MEMS 3-axis accelerometer Altimeter	HR zones	Fitbit app	NO
Fitbit Ionic	HR	Optical	wrist	athlete	HR on watch	GPS/GLONASS MEMS 3-axis accelerometer Barometric altimeter	HR zones	Fitbit app	NO
Garmin Fenix 5	HR	Optical	wrist	athlete	HR on watch	GPS/GLONASS/Galileo Accelerometer Gyroscope Barometric altimeter Compass Thermometer	HR zones and HR alerts	Garmin Connect Mobile app	NO
Garmin Forerunner 235	HR	Optical	wrist	athlete	HR on watch	GPS/GLONASS Accelerometer Thermometer	HR zones and HR alerts	Garmin Connect Mobile app	NO
Garmin Forerunner 305	HR	Capacitive electrode	wrist chest	athlete	HR on watch	GPS	HR zones and HR alerts	Garmin Express on computers	NO
Garmin Venu Sq	HR	Optical	wrist	athlete	HR on watch	GPS/GLONASS/Galileo Accelerometer Compass Thermometer Pulse OX blood oxygen saturation monitor	HR zones and HR alerts	Garmin Connect Mobile app	NO
Garmin Vivosmart HR	HR	Optical	wrist	athlete	HR on watch	Accelerometer Barometric altimeter	HR zones and HR alerts	Garmin Connect Mobile app	NO
Hexoskin shirt	1-lead ECG HR	Capacitive electrode	chest	athlete	HR and ECG on smartphone	RIP 3-Axis accelerometer	HR zone, HRV, HR maximum and HR at rest, QRS events	Hexoskin app	NO
Jabra Elite Sport Earbuds	HR	Optical	Ear	athlete	HR on smartphone	-	Cardio performance	Jabra Sport Life app	NO
Adidas Smart sports bra	HR	HR sensing fabric	chest	athlete	-	-	-	-	NO
PulseOn	ECG HR	Capacitive electrode Optical	wrist	doctor	-	-	Notification for irregular rhythm	PulseOn app	NO
Scosche Rhythm+	HR	Optical	Arm	athlete	HR on the receiving device	-	-	Compatible with >200 fitness apps	NO
Suunto Spartan Sport watch	HR	Optical	wrist	athlete	HR on watch	GPS/GLONASS Accelerometer Altimeter	HR zones	Suunto app	NO
Kardia AliveCor	ECG HR	Dry electrode	-	athlete doctor	HR and ECG on smartphone	-	Sinus rhythm, AF, bradycardia, tachycardia	Kardia app	FDA-cleared
Kardia 6L AliveCor	ECG HR	Dry electrode	-	athlete doctor	HR and ECG on smartphone	-	Sinus rhythm, AF, bradycardia, tachycardia	Kardia app	FDA-cleared
Motiv Ring	HR	Optical	finger	athlete	HR on smartphone	3-axis accelerometer	-	Motiv 24/7 Smart Ring app	NO
Polar H10 chest strap	HR	Capacitive electrode	chest	athlete	HR on the receiving device	-	HR zones and HR alerts	Polar Beat app Polar Flow app	NO
Polar H7 chest strap	HR	Capacitive electrode	chest	athlete	HR on the receiving device	-	HR zones and HR alerts	Polar Beat app Polar Flow app	NO
Polar OH1	HR	Optical	forearm	athlete	HR on the receiving device	-	HR zones and HR alerts	Polar Beat app Polar Flow app	NO
Polar S810	HR	Capacitive electrode	wrist + chest	athlete	HR on watch	-	HR zones and HR alerts	-	NO
Polar S810i	HR	Capacitive electrode	wrist + chest	athlete	HR on watch	-	HR zones and HR alerts	-	NO
Polar T31^TM^ Coded band	HR	Capacitive electrode	chest	athlete	HR on the receiving device	-	HR zones and HR alerts	Polar Beat app Polar Flow app	NO
Polar Pro sensor	HR	Capacitive electrode	chest	athlete coach	HR on the receiving device	GPS Accelerometer Gyroscope Compass	HR zones and HR alerts	PC software PDA software (for online monitoring)	NO
Polar V800	HR	Capacitive electrode	wrist + chest	athlete	HR on watch	GPS Accelerometer	HR zones and HR alerts	Polar Flow app	NO
Polar Vantage M	HR	Optical	wrist	athlete	HR on watch	GPS/GLONASS/Galileo Accelerometer	HR zones	Polar Flow app	NO
Polar Vantage V2	HR	Optical	wrist	athlete	HR on watch	GPS/GLONASS/Galileo Accelerometer Barometer Compass	HR zones	Polar Flow app	NO
Polar Ignite sport watch	HR	Optical	wrist	athlete	HR on watch	GPS/GLONASS/Galileo Accelerometer	HR zones	Polar Flow app	NO
Samsung Galaxy Watch 3	HR	Optical	wrist	athlete	HR on watch	GPS/GLONASS/Galileo Accelerometer Gyroscope Barometer	Normal and irregular sinus rhythm	Samsung Health Monitor app	NO
Berlei sports bra	HR	Capacitive electrode	chest	athlete	-	-	-	-	NO
Sensoria fitness sports bra + HRM	HR	Capacitive electrode	chest	athlete	HR on smartphone	-	-	Sensoria HRM Sensoria Fitness mobile app	NO
TomTom Spark 3	HR	Optical	wrist	athlete	HR on watch	GPS Accelerometer Barometer Compass	HR zones	TomTom Sports app	NO
TomTom Spark Cardio	HR	Optical	wrist	athlete	HR on watch	GPS Accelerometer Compass	HR zones	TomTom Sports app	NO

GPS = Global Positioning System; GLONASS = Global Navigation Satellite System; MEMS = micro electro-mechanical systems; RIP = respiratory inductance plethysmography.

In the present review, only devices satisfying the eligibility criteria were considered. Consequently, some device versions of considered brands (e.g., Applewatch series 6) or devices of unconsidered brands (e.g., Huawei) may not appear in our tables. Wristbands (23/38, 61% in this study) are becoming increasingly popular and investigated [21,23,24,25,27,28,29,30,53], in particular smart watches, which are fashion commodities offering purposes beyond visual appeal that in many cases provide users with a plethora of health-related data [11]. The user’s choice of which device to pick also depends on activity type. Specifically, a chest strap (e.g., Polar H10) is recommended for precise monitoring, because it provides better accuracy even in high-intensity training [21,23,26,28]. Although chest bands offer greater accuracy in HR monitoring and cost less, wristbands are more desirable, because of their multifunctionality and comfort. In sport, sensor-embedded equipment and smart textiles are also exploited to enable users to have high-quality signals without hindering any movement [31,44,55].

Sensor placement depends on sport, athletic movement or external factors, such as presence of possible concussions/contacts [10]. Further different sports of application and different types of users define the design of wearable and portable devices and the components needed. Some devices embed other sensors or exploit the ones embedded in the receiving device, usually a smartphone. Further components and measures usually are breathing sensors to derive respiration rate; accelerometers and gyroscope to derive body orientation, activity, steps, cadence, calories burned and sleep data; altimeters to derive floors climbed; and positioning systems based on satellites to derive distance covered and speed.

Of note, 11 devices were found to be discontinued and one recalled, namely, Fitbit Ionic, whose battery could overheat, posing a burn hazard to consumers. The devices still in production can be connected to another system via a specific application to display the data obtained during acquisition. Some wrist devices present a monitor that allows one to check data in real time. Among all the devices, the Kardia by AliveCor [22,45] stood out for its target user, the clinician, and is the only one FDA-cleared. This portable device is able to detect atrial fibrillation, bradycardia, tachycardia and normal heart rhythm. Monitoring for heightened risk of atrial fibrillation seems needed amongst endurance athletes [56,57,58]. Most others, on the other hand, estimate the user’s maximum HR based on actual HR zone, i.e., a set range of heart beats per minute. Many runners and other athletes are using HR zones to measure and increase their cardiovascular strength and improve their level of fitness.

### 4.1. Validation Studies

A rigorous assessment of validity should be in the mutual interests of manufacturers, scientific institutions, and consumers in order to judge whether a wearable device for assessment of HR is useful and performs with satisfactory accuracy.

To validate wearable devices against standard apparatus such as ECG through multiple-lead channels or simple chest straps consisting of two electrodes is strongly recommended. The 12-lead ECG is the current gold-standard reference; however, several studies used as a reference device a chest strap recorder if the device needed to be validated in dynamic conditions, such as sport activities. High-quality HR data for the Polar H7 was demonstrated by Pasadyn et al. [24] and Gillinov et al. [27], who compared the acquired HR to those acquired by clinical instrumentation and reported Lin’s concordance correlation coefficients of r_c_ = 0.98 and r_c_ = 0.99, respectively. In recent studies, the Polar H7 was superseded by the later model Polar H10 [21,23,24,25,26,27,28], which for incremental exercise shows a Lin’s concordance correlation coefficient of r_c_ = 0.93 when comparing its ECG to a 12-lead ECG [26].

The validation process has been performed on many wearable devices, most of them wrist-worn devices based on optical PPG technology. Among them, the Apple Watch III proved to be the optimal choice for assessing HR during high-speed running (r_c_ = 96) [24].

Accuracy and precision of the Polar Vantage V2 and Garmin Venu Sq have been analyzed during swimming, providing unsatisfactory results: water and arm movement acted as relevant interference inputs. Therefore, for monitoring of HR of swimming athletes, use-specific wearable devices are recommended [21].

Overall, these findings highlight that the validation process provides heterogeneous results due to the different types of activities and the intensity of these. Variability in the expression of the metrological characteristics also emerged, e.g., referring to accuracy, some authors used mean absolute percentage error (MAPE), others Pearson’s coefficient (r) or Lin’s concordance correlation coefficient (r_c_). As the data are quite inhomogeneous, they can be scarcely compared. Moreover, the number of wearable devices is rapidly growing, and companies and consumers would benefit from guiding standardized protocols.

Validation studies are important to guide device design since the effect of sensor technology, sensor wear location, and physical activity may affect the performance of the device [21,23,26,28]. Additionally, chest straps based on capacitive sensor technologies are precise and provide good accuracy even in high-intensity training, with breathing interference mainly affecting the measurement. Wristbands and smart watches based on optical sensor technologies are affected by artifact movements and usually underestimate HR. Acquisitions by smart watches are particularly affected by their multifunctionality (they may work as ECG recorders, watches, phones, etc. simultaneously). Sensor-embedded equipment and smart textiles may enable users to have high-quality signals without hindering any movement, especially in contact sports [31,44,55], hence the development of a validation protocol for wearable devices measuring cardiac signals is desirable. With this common aim, six universities and one industrial partner joined to present a set of guidelines to obtain more comparable data. The statement focused on six standardized domains: target population, criterion measure, index measure, testing conditions, data processing and statistical analysis [59].

### 4.2. Clinical Studies

Clinical studies were conducted for various sports using different type of wearables. Among the various devices, the Polar Pro Sensor was recurrent (5/35) [34,35,36,37,38]. This chest strap is included in the Polar Team Pro system and allows real-time HR monitoring of multiple athletes simultaneously. Therefore, this system is widely used in team sports, such as basketball, football, and volleyball [34,35,36,37,38]. This technology allows coaches to track athletes’ parameters during training session and competition.

Two other devices of remarkable interest are BioHarness 3.0 by Zephyr and Kardia by AliveCor. The BioHarness 3.0 by Zephyr was used in a great variety of sports [42,43,49,50,51,52] to evaluate the health status of athletes based on HR variability [42] and to characterize ECG during the pre-exercise phase [43], providing reference values for future diagnosis. ECG has also been acquired using a portable device called AliveCor Kardia, which helps the diagnosis of arrhythmias during exercise in athletes [45,56,57].

Only the AliveCor Kardia was FDA-cleared, whereas all other devices are not clinically approved and thus cannot be used for cardiac diagnosis. Typically, wearable sensors provide a reduced number of ECG leads, which do not necessarily match with one of the 12 standard ECG leads. Additionally, acquisition settings of these sensors do not match the typically strict protocols followed in the clinical setting [43]. Consequently, they cannot be used for diagnoses: considering that the normal reference values used in clinics are defined considering the standard 12-lead ECG, measured ECG values by wearable sensors should not be considered to evaluate the athlete’s health [43]. Validation studies [21,22,23,24,25,26,27,28,29,30,31] and a recent study on the development of normal reference values for ECG acquired through wearable chest straps in the pre-exercise phase [43] can play a pivotal role in the implementation of wearable devices in clinical practice.

### 4.3. Development Studies

Among the development studies, only one focused on proposing an open-source database that can be useful for new studies. The database is called Sport DB and consists of 126 cardiorespiratory datasets acquired through the chest strap BioHarness 3.0 by Zephyr from athletes practicing 10 different sports [50].

As for the algorithms, each study was conducted with a different aim and different devices were used. The Hexoskin biometric compression shirt was used in [55] to demonstrate the capability of the microlocation-specific biometric system. The BioHarness 3.0 was used in [49] for the proposal of a tool called CaRiSMA 1.0, in [51] for the presentation of a predictive analytics framework for predicting soccer players’ performance data, and in [52] for the development of an algorithm for automatic detection of training phases. The Polar H10 was used in [46] for indirect estimation of breathing rate from HR acquired by the chest belt during running. The Polar T31TM coded band was used in [47] for the proposal of an intelligent data analytics system for swimmer performance and in [48] for the proposal of a novel system that allows the technical staff to monitor and analyze the swimmer’s inertial and bio-signals in real time. The Samsung Galaxy Watch 3 in [53] for precise real-time HR monitoring during high-intensity physical exercises and the Garmin Forerunner 305 [54] device to estimate the intensity of activities were used.

The lack of databases suggests that future studies should develop open-source databases with the goal of making more information regarding sports activity available. Such data could be useful for further studies, such as the development of new automatic algorithms.

### 4.4. Related Works

This being a review study, Table 1, Table 2 and Table 3 do not report results from other review studies on the topic [2,60,61,62,63,64,65,66,67,68]. However, their qualitative analysis may be useful to highlight the strengths of this study. Li at al. [2] evaluated the applicability of wearable devices in sport science to increase training performance and focused on the modality of monitoring real-time physiological and movement parameters during training and competitive sports. Rao et al. [60] focused on the role of only wearable devices to diagnose and monitor cardiovascular disease in sport cardiology. Seshadri et al. [61] focused on the clinical translation of biomedical sensors for sports medicine. Other review papers focused on novel noncommercial sensing technologies (sensing textiles, flexible sensors, and sensor-embedded equipment) [62,63]. Other reviews focused only on specific sport activities [64,65] or on the validation of specific devices [66,67,68].

Differently from the abovementioned reviews, the present review focused on applications of not only wearable but also portable devices in training and cardiovascular monitoring. Moreover, our work investigated only commercial devices (i.e., consolidated technology) and highlighted their limits to support design of future innovative technology. Finally, our work represents a comprehensive (not specific) overview of the use of wearable and portable devices for cardiac signal acquisition and related tool validation while practicing sport.

## 5. Conclusions

Wearable and portable devices have been the leading technologies in sport trends in the last 11 years and represent the future of sport industry development. Results from clinical studies highlighted that wearable devices are crucial to improve athletes’ performance and to prevent adverse cardiovascular events. At the same time, the need for standardized validation of these technologies emerged. Future development of standardized data-acquisition protocols, signal processing procedures specifically designed for sport, and sport-oriented software applications will cover a key role in the clinical interpretation of data acquired through wearable and portable devices. This innovative approach will lead to athlete-centered monitoring, which will allow adaptation of the training regime for maximizing performance and minimizing cardiovascular risk.

## Data Availability

Not applicable.

## Supplementary Materials

The following supporting information can be downloaded at https://www.mdpi.com/article/10.3390/s23063350/s1. Search queries: “Search_Query.pdf”, Sources of specification of wearable and portable devices “Specification_Device_Sources.pdf”.
