# Supplementary material for: Wearable and Portable Devices for Acquisition of Cardiac Signals while Practicing Sport: A Scoping Review
Source: Sensors (Basel). 2023 Mar 22;23(6):3350. doi: 10.3390/s23063350 (PMC10055735; doi:10.3390/s23063350)
Supplement: Supplementary file 1 [file sensors-23-03350-s001.zip › Search_Query.pdf]

Search query in Pubmed

(("sport*"[Title/Abstract] OR "athlet*"[Title/Abstract]) AND (("wearable"[Title/Abstract] OR "portable"[Title/Abstract]) AND ("sensor*"[Title/Abstract] OR "electronic*"[Title/Abstract] OR "device*"[Title/Abstract])) AND ("heart rate"[Title/Abstract] OR electrocardio*"[Title/Abstract]))

Search query in Scopus

TITLE-ABS ((sport* OR athlet*) AND ((wearable OR portable) AND (sensor* OR electronic* OR device*)) AND (heart rate OR electrocardio*))

Search query in Web of science

TI= ((sport* OR athlet*) AND ((wearable OR portable) AND (sensor* OR electronic* OR device*)) AND (heart rate OR electrocardio*)) OR AB= ((sport* OR athlet*) AND ((wearable OR portable) AND (sensor* OR electronic* OR device*)) AND (heart rate OR electrocardio*))

The document search was conducted on February 9, 2023.
