# Supplementary material for: Wearable and Portable Devices for Acquisition of Cardiac Signals while Practicing Sport: A Scoping Review
Source: Sensors (Basel). 2023 Mar 22;23(6):3350. doi: 10.3390/s23063350 (PMC10055735; doi:10.3390/s23063350)
Supplement: Supplementary file 1 [file sensors-23-03350-s001.zip › Specification_Device_Sources.pdf]

Table S1. reports the manual / scientific literature or manufacturer website, containing the specification of devices.

Table S1. Manual / scientific literature or manufacturer website, containing the specification of devices. Not available information was reported as "-".

Device	Manual / Scientific literature	Manufacturer website
Apple Watch I	-	https://support.apple.com/kb/sp745?locale=en_US
Apple Watch III	-	https://support.apple.com/kb/sp766?locale=it_IT
BioHarness 3.0 Zephyr	Zephyr™ BioHarness 3.0 User Manual https://www.zephyranywhere.com/media/download/bioharness3-user-manual.pdf	-
Fitbit Blaze	Fitbit Blaze User Manual https://help.fitbit.com/manuals/manual_blaze_en_US.pdf	https://www.fitbit.com/pl/shop/blaze
Fitbit Charge 3	Fitbit Charge 3 User Manual https://staticcs.fitbit.com/content/assets/help/manuals/manual_charge_3_en_US.pdf	https://help.fitbit.com/en_US/ionic.htm
Fitbit Ionic	Fitbit Ionic User Manual https://staticcs.fitbit.com/content/assets/help/manuals/manual_ionic_en_US.pdf	-
Garmin Fenix 5	fēnix® 5/5S/5X Plus Owner's Manual https://www8.garmin.com/manuals/webhelp/fenix5plus/EN-US/fenix_5_Plus_Series_OM_EN-US.pdf	https://www.garmin.com/it-IT/p/552982
Garmin Forerunner 235	Forerunner® 230/235 Owner's Manual https://www8.garmin.com/manuals/webhelp/forerunner230/EN-US/Forerunner_230_235_OM_EN-US.pdf	https://www.garmin.com/it-IT/p/529988
Garmin Forerunner 305	Forerunner205-305 https://static.garmin.com/pumac/FR_205_305_OM_IT.pdf	https://www.garmin.com/it-IT/p/349
Garmin Venu Sq	Venu® Sq Owner's Manual https://www8.garmin.com/manuals/webhelp/GUID-1C3C7630-B695-44C3-AF56-949C1D4889FB/EN-US/Venu_Sq_OM_EN-US.pdf	https://www.garmin.com/it-IT/p/707174
Garmin Vivosmart HR	Vívosmart® HR/HR+ Owner's Manual https://www8.garmin.com/manuals/webhelp/vivosmarthr/EN-US/vivosmart_HR_and_HR_GPS_OM_EN-US.pdf	https://www.garmin.com/it-IT/p/531166
Hexoskin	Hexoskin Product Specifications	https://www.hexoskin.com/
Jabra Elite Sport Earbuds	EN Jabra Elite Sport User Manual RevF https://www.jabra.com/_/media/Jabra_VXi_Product-Documentation/Jabra-Elite-Sport/User-manual/RevF/EN-Jabra-Elite-Sport-User-Manual-RevF.pdf	-
Adidas HR monitor sport bra	Navalta, J.W.; Ramirez, G.G.; Maxwell, C.; Radzak, K.N.; McGinnis, G.R. Validity and Reliability of Three Commercially Available Smart Sports Bras during Treadmill Walking and Running. *Scientific Reports* **2020**, *10*, 7397. DOI: 10.1038/s41598-020-64185-z.	-
PulseOn	-	https://pulseon.com/
Scosche Rhythm+	-	https://www.scosche.com/rhythm-plus-heart-rate-monitor-armband

Continue Table S1 Manual / scientific literature or manufacturer website, containing the specification of devices. Not available information was reported as "-".

Device	Manual / Scientific paper	Website
Kardia AliveCor	-	https://www.alivecor.com/products/
Kardia 6L AliveCor	-	https://www.alivecor.com/products/
Motiv Ring	-	https://www.mymotiv.com/
Polar H10	Polar H10 User manual https://support.polar.com/e_manuals/h10-heart-rate-sensor/polar-h10-user-manual-english/manual.pdf	https://www.polar.com/it/sensors/h10-heart-rate-sensor
Polar H7	Polar_H7_Heart_Rate_Sensor_accessory_manual_English https://support.polar.com/e_manuals/H7_Heart_Rate_Sensor/Polar_H7_Heart_Rate_Sensor_accessory_manual_English__.pdf	-
Polar OH1	Polar OH1 User Manual https://support.polar.com/e_manuals/OH1/Polar_OH1_user_manual_English/manual.pdf	https://www.polar.com/it/sensors/oh1-optical-heart-rate-sensor
Polar S810	Manual S810 https://support.polar.com/e_manuals/S810/179291%20Manual%20S810%20GBR%20A.pdf	-
Polar S810i	Polar-s810i-user-guide-english https://support.polar.com/e_manuals/s810i/polar-s810i-user-guide-english.pdf	-
Polar T31 coded™	Getting_Started_Guide_for_Polar_T31C_English https://support.polar.com/e_manuals/T31C/Getting_Started_Guide_for_Polar_T31C_English.pdf	https://www.polar.com/en/products/accessories/T31_coded_transmitter
Polar Pro sensor	Polar Team Pro con GPS https://support.polar.com/e_manuals/Team_Pro/Polar_Team_Pro_user_manual_English/manual.pdf	https://www.polar.com/ae-en/b2b_products/team-pro
Polar V800	Polar V800 User Manual https://support.polar.com/e_manuals/V800/Polar_V800_user_manual_English/manual.pdf	-
Polar Vantage M	Polar Vantage M User Manual https://support.polar.com/e_manuals/vantage-m/polar-vantage-m-user-manual-english/manual.pdf	https://www.polar.com/en/vantage/m
Polar Vantage V2	Vantage V2 User Manual https://support.polar.com/e_manuals/vantage-v2/polar-vantage-v2-user-manual-english/manual.pdf	https://www.polar.com/en/vantage/v2
Polar Ignite sport watch	Vantage Ignite Manual https://support.polar.com/e_manuals/ignite/polar-ignite-user-manual-english/manual.pdf	https://www.polar.com/en/ignite
Samsung Galaxy Watch 3	-	https://www.samsung.com/us/support/downloads/?model=N0054172
Berlei sport bra	Navalta, J.W.; Ramirez, G.G.; Maxwell, C.; Radzak, K.N.; McGinnis, G.R. Validity and Reliability of Three Commercially Available Smart Sports Bras during Treadmill Walking and Running. *Scientific Reports* **2020**, *10*, 7397. DOI: 10.1038/s41598-020-64185-z.	-
Sensoria fitness sports bra + HRM	-	https://store.sensoriafitness.com/sports-bra-black/
Suunto Spartan Sport	-	https://www.suunto.com/Support/sports-watches-support/suunto-spartan-sport/
TomTom Spark Cardio	-	https://www-preprod-origin.tomtom.com/it_it/navigation/
TomTom Spark 3	-	https://www-preprod-origin.tomtom.com/it_it/navigation/
